# CD72/CD100 and PD-1/PD-L1 markers are increased on T and B cells in HIV-1^+^ viremic individuals, and CD72/CD100 axis is correlated with T-cell exhaustion

**DOI:** 10.1371/journal.pone.0203419

**Published:** 2018-08-30

**Authors:** Rafael Correa-Rocha, Jacobo Lopez-Abente, Carolina Gutierrez, Verónica Astrid Pérez-Fernández, Adrián Prieto-Sánchez, Santiago Moreno-Guillen, María-Ángeles Muñoz-Fernández, Marjorie Pion

**Affiliations:** 1 Immuno-Regulation Laboratory, Gregorio Marañón University General Hospital, Gregorio Marañón Health Research Institute (IiSGM), Madrid, Spain; 2 Department of Infectious Diseases, Hospital Ramón y Cajal, Alcalá de Henares University, Instituto Ramón y Cajal de Investigación Sanitaria (IRYCIS), Madrid, Spain; 3 Immuno-Biology Molecular Laboratory, Gregorio Marañón University General Hospital, Gregorio Marañón Health Research Institute (IiSGM), Spanish HIV HGM BioBank, Madrid, Spain; 4 Networking Research Center on Bioengineering, Biomaterials and Nanomedicine (CIBER-BBN), Madrid, Spain; University Hospital Zurich, SWITZERLAND

## Abstract

In our work, we analyzed the role of the CD100/CD72 and PD-1/PD-L1 axes in immune response dysfunction in human immunodeficiency virus (HIV)-1 infection in which high expressions of PD-1 and PD-L1 were associated with an immunosuppressive state via limitation of the HIV-1-specific T-cell responses. CD100 was demonstrated to play a relevant role in immune responses in various pathological processes and may be responsible for immune dysregulation during HIV-1 infection. We investigated the function of CD72/CD100, and PD-1/PDL-1 axes on T and B cells in HIV-infected individuals and in healthy individuals. We analyzed the frequencies and fluorescence intensities of these four markers on CD4^+^, CD8^+^ T and B cells. Marker expressions were increased during active HIV-1 infection. CD100 frequency on T cells was positively associated with the expression of PD-1 and PD-L1 on T cells from HIV-infected treatment-naïve individuals. In addition, the frequency of CD72-expressing T cells was associated with interferon gamma (IFN-γ) production in HIV-infected treatment-naïve individuals. Our data suggest that the CD72/CD100 and PD-1/PD-L1 axes may jointly participate in dysregulation of immunity during HIV-1 infection and could partially explain the immune systems’ hyper-activation and exhaustion.

## Introduction

Dysregulation of HIV-specific T and B-cell responses is the principal cause for the lack of control of HIV replication. Chronic infection with the persistent presence of viral antigens gives rise to B- and T-cell exhaustion, which is characterized by loss of proliferative capacity and effector functions [1, 2]. Negative regulatory pathways (such as the PD-1/PD-L1 axis) under physiological conditions play an important role in maintaining peripheral tolerance and preventing excessive immune activation [3, 4]. Nonetheless, excessive activation of negative regulatory pathways induces immune exhaustion in part via the PD-1/PD-L1 axis. The PD-1/PD-L1 axis was identified as the major regulator of T-cell exhaustion during chronic HIV/SIV infection and appears to be responsible for the dysfunction of HIV-specific CD8^+^ T cells [5–10]. Increased PD-1 was also associated with T-cell exhaustion in HIV/*Mycobacterium tuberculosis* co-infection and was associated with senescence and activation markers on mucosal-associated invariant T cells during HIV and hepatitis C virus (HCV) infection [11–13]. PD-1 expression is induced on CD4^+^, natural killer (NK) T-cell subsets, B cells, monocytic cells, and most notably on the surface of CD8^+^ T cells upon activation during HIV-1 infection [7, 13, 14]. PD-L1 is constitutively expressed on B cells, dendritic cells (DCs), macrophages and T cells, and it is also upregulated upon activation [15]. The PD-L1 expression levels on DCs and monocytes positively correlate with viral load (VL) in HIV-1^+^ individuals [16]. The PD-L1 expression was also observed at the surface of T cells in HIV-1^+^ individuals, and blockade of PD-L1 was shown to induce higher proliferation of specific anti-Gag T cells [17]. Altogether, these data suggest that the PD-1/PD-L1 pathway plays an important role in exhaustion of anti-viral CD8^+^ T cells during chronic HIV-1 infection. Nonetheless, little is known about B-cell dysregulation since B cells may bear PD-1 and PD-L1 markers on their surfaces. However, PD-1 induces negative regulation of B-cells activation [18]. Therefore, PD-1 and PD-L1 could have an antagonist role.

In HIV-1 infection, immune cell dysregulation is multifactorial, and recent publications indicate that CD72/CD100 may play a relevant role in immune regulation [19–21]. It was demonstrated that CD100, which is constitutively expressed on T cells, and CD72 expression could be upregulated on the surface of T cells upon activation [22]. CD72 is essentially expressed at the surface of antigen presenting cells (such as B cells), but it was also observed at the surface of Treg cells in which CD72 is involved in Foxp3^+^CD4^+^ Treg cells expansion [23]. CD72 on B cells induces dual effects that can be explained by its association with positive and negative signaling molecules [24, 25]. Indeed, it was observed that CD72 has not only been implicated in naïve B-cell differentiation suppression and reduced survival of mature B cells but also participates in IL-10 production by regulatory B cells [23]. More recently, it was described that CD72/CD100 interaction was also required for T-cell proliferation and that CD100 up-regulation at the surface of B cells was associated with B-cell activation and differentiation [22, 26]. CD100-associated signaling rescues B cells from apoptosis and causes B-cell proliferation and survival [27–29]. In the context of HIV infection, it was observed that a decrease in CD100 expression at the surface of CD8^+^ T cells was linked to reduced effector functions that render T cells incapable of optimally responding to pathogens [19]. Also, in the context of HCV infection, it was observed that CD8^+^ T cells expressing CD100 were able to induce the production of interferon alpha (IFNα) through the CD72/CD100 axis [30]. Thus, the CD72/CD100 axis appears to have an important role in the T- and B-cell functions. However, it is still unclear what role the CD72/CD100 axis has during normal immune responses and HIV-1 infections.

We showed a general dysregulation in which CD100-expressing CD4^+^ T cells correlated with exhaustion markers such as PD-1 on CD4^+^ and CD8^+^ T cells and the negative regulatory marker PD-L1 on CD4^+^ and CD8^+^ T cells in HIV-infected treatment-naïve individuals. These correlations could be associated with a possible attempt by the immune system to limit T-cell activation and proliferation. PD-L1-expressing B-cell frequencies were associated with the frequencies of CD72-expressing CD4^+^ and CD8^+^ T cells in healthy individuals that were lost in HIV-1^+^ individuals and, could be related to the loss of the suppressive ability over the activated T cells in viremic patients. Moreover, a clear correlation between the intensity of CD72 expression at the surface of CD8^+^ T cells and the ability of T cells to express IFN-γ was observed. Altogether, these outcomes indicate a link between the regulatory markers on T and B cells, thereby confirming the role of HIV-1 infection in dysregulating immune system homeostasis.

Interestingly, little is known about the relationships between CD72/CD100 and PD-1/PD-L1 axes in the context of HIV-1 infection. The relation between T and B cells is multifactorial, and it is believed that this may have important implications for disease progression. Therefore, the study of cell surface marker dysregulation that is involved in negative and positive signaling of the immune response is crucial to better understanding of HIV-1 infection pathogenesis.

## Materials and methods

### Participants

All individuals were recruited under a protocol accepted by the Clinical Ethics Committee of the Ramón y Cajal Hospital (reference number: RyC-419-14) and the Gregorio Marañón Hospital (reference number: HGUGM-04/2014) according to the principles described in the Declaration of Helsinki (2013). Data analyses were based on anonymized routine clinical data. Written informed consent for study participation was obtained from all individuals. The research was conducted between December 2014 and December 2016. All samples were processed within one hour after sample reception on the day of blood extraction. We studied three groups of individuals: i) HIV-infected treatment naïve individuals that were newly diagnosed. Peripheral blood samples were collected before they received antiretroviral treatment and presented with detectable viral loads (VL) in plasma (group VL; superior to 50 copies/ml of plasma); ii) treated-HIV-1^+^ individuals who received antiretroviral treatment and presented with undetectable VL in plasma (group uVL; inferior to 50 copies/ml of plasma); and iii) healthy individuals who were not infected by HIV-1 used as the control group (Table 1). Positivity diagnosis for HIV-1 was done by the use of enhanced ELISA assays that detected both HIV antibodies and antigens (fourth generation assay) and permits earlier detection of HIV seroconversion followed by western blot confirmation.

**Table 1:** Characteristics of HIV-1^+^-infected and healthy individuals enrolled in the study and characterization of T and B cells.

	**VL (i)**	**uVL (ii)**	**Control (iii)**		**Control vs VL**	**Control vs uVL**	**VL vs uVL**
	Median [Q1-Q3] *	Median [Q1-Q3] *	Median [Q1-Q3] *	**Test p**	**p**	**p**	**p**
n	13	14	12		-	-	-
Gender (male; %)	93	75	85	0.453 ¥	-	-	-
Age (years)	**36.0** [25.8–50.5]	**41.5** [35.0–53.0]	**39.0** [24.0–46.5]	0.453 ¶	-	-	-
Years Diagnosed	-	**5.0** [3.0–9.5]	-	-	-	-	-
Years under HAART	-	**4.0** [2.7–8.2]	-	-	-	-	-
CD4 Nadir (%)	**19.6** [9.5–30.7]	**14.5** [8.9–19.9]	-	-	-	-	0.301 **§**
CD4 Nadir (cells count/μl)	**380.8** [66.7–517.2]	**244.5** [122.6–349.2]	-	-	-	-	0.681 **§**
Plasma viral load (copies/ml)	**63096** [1000–1258925]	ND	-	-	-	-	-
Years with undetectable viral load	-	**3.5** [2.0–7.0]	-	-	-	-	-
	**VL (i)**	**uVL (ii)**	**Control (iii)**	**Krukal-Wallis**	**Control vs VL**	**Control vs uVL**	**VL vs uVL**
	**Median [Q1-Q3]**	**Median [Q1-Q3]**	**Median [Q1-Q3]**	**Test p** ¶	**p** ⱡ	**p** ⱡ	**p** ⱡ
% Gated in CD19+ cells		^ ^					
Total B cells	**6.0** [4.1–8.8]	**8.7** [6.2–13.2]	**10.3** [9.0–11.8]	**0.008**	**0.009**	0.912	0.105
Memory Ɨ	**28.7** [7.4–41.5]	**15.0** [9.7–15.7]	**31.1** [23.1–42.9]	0.496	-	-	-
Naive Ɨ	**11.1** [3.3–62.1]	**79.3** [5.0–80.8]	**47.5** [9.3–64.6]	0.496	-	-	-
Plasmablast Ɨ	**2.35** [0.48–7.17]	**0.09** [0.08–0.25]	**0.30** [0.22–0.51]	**0.015**	0.123	0.156	0.084
% Gated in CD4+ T cells							
Total CD4+ T cells	**24.3** [8.2–35.1]	**31.0** [21.1–36.1]	**42.8** [37.5–46.6]	**<0.001**	**<0.001**	**<0.001**	0.732
Activated Ɨ	**8.2** [4.4–25.3]	**3.7** [2.3–5.1]	**2.0** [1.6–2.6]	**<0.001**	**<0.001**	0,072	**0.033**
Naive Ɨ	**23.4** [9.8–34.3]	**40.0** [28.9–44.1]	**29.7** [24.6–44.9]	**0.032**	0.219	1.000	**0.036**
Memory Ɨ	**53.1** [44.8–73.8]	**38.9** [32.1–53.2]	**40.3** [31.3–49.1]	**0.021**	**0.021**	1.000	**0.048**
% Gated in CD8+ T cells							
Total CD8+ T cells	**48.9** [38.3–54.1]	**30.6** [23.6–43.0]	**18.8** [15.3–22.7]	**<0.001**	**<0.001**	**0.003**	**0.021**
Activated Ɨ	**22.0** [14.0–39.6]	**5.9** [2.3–8.3]	**2.8** [1.1–5.8]	**<0.001**	**<0.001**	0.594	**<0.001**
Naive Ɨ	**6.5** [3.3–13.9]	**18.4** [9.3–34.1]	**33.3** [28.7–40.9]	**<0.001**	**<0.001**	**0.042**	**0.027**
Memory Ɨ	**32.6** [25.6–48.7]	**21.7** [12.4–28.0]	**20.9** [10.7–30.1]	**0.007**	**0.012**	1.000	**0.015**
Ratio CD4/CD8	**0.54** [0.15–0.77]	**0.85** [0.59–1.13]	**2.25** [1.80–2.71]	**<0.001**	**<0.001**	**<0.001**	0.087

(i)VL: HIV-1^+^ treatment-naïve individuals; (ii) uVL treated-HIV-1^+^ individuals; and (iii) Control: healthy individuals. Q1: quartile 1 and Q3: quartile 3. ND: not detectable. We determined T and B cell phenotypes in HIV-1^+^ and healthy individuals. Statistical analyses were performed with Pearson’s chi-squared test (¥); Kruskal-Wallis test (¶); non-parametric Mann–Whitney U test when comparing VL and uVL groups (§) or paired Mann-Whitney U test with Bonferroni correction when comparing the three groups of individuals (ⱡ): Results for T and B cell characterization were represented as median [Q1-Q3] excepted for Gender (% of male, *). Ɨ Gated on Total Cells. ± SEM. Statistically significant: *p* <0.05.

### Direct labeling of whole blood and intracellular staining

Whole blood was surface labeled, lysed, and analyzed by flow cytometry (S1 Table presents the list of antibodies used in this study and S1–S3 Figs). For intracellular cytokine labeling, 1×10^6^ of freshly isolated peripheral blood mononuclear cells (PBMCs) were cultured for 5 h with phorbol 12-myristate 13-acetate (PMA, Sigma-Aldrich; 10ng/ml) and ionomycin (Sigma-Aldrich; 0.25μg/mL) at 37°C, 5% CO_2_; GolgiStop was added after the first 1 h of culture. Cells were then surface labeled, washed, intracellularly labeled using the Fixation/Permeabilization Solution Kit (BD Biosciences), and then analyzed by flow cytometry using a Gallios cytometer (Beckman Coulter). Data were analyzed using Kaluza Analysis software (Beckman Coulter). The Fluorescence Minus One Control (FMO) was used to identify and gate cells in the context of IFNγ, PD-L1; PD-1; CD72 and CD100 labeling (S1–S3 Figs). Naïve CD4^+^ or CD8^+^ T cells were defined as CD45RA^+^CD27^+^ (S1 Fig). Memory CD4^+^ or CD8^+^ T cells were defined as CD45RO^+^ (S1 Fig) and activated CD4^+^ or CD8^+^ T cells were defined as CD45RO^+^HLA-DR^+^. Naïve and memory B cells, and plasmablasts were defined as CD19^+^IgD^+^CD27^-^; CD19^+^CD27^+^ and CD19^+^CD38^hi^CD24^-^, respectively (S1 Fig).

### Statistical analysis

Comparisons of cell populations between healthy individuals and treatment-naïve HIV-1^+^ individuals or treated-HIV-1^+^ individuals were performed using the non-parametric Kruskal-Wallis ANOVA test, followed by Mann-Whitney U tests with a Bonferroni correction for multiple testing when conditions presented significance (p<0.05 in Kruskal Wallis test). For comparison between two the groups, paired Mann-Whitney U tests were used. For gender analysis, Pearson’s chi-squared test was used. The statistical correlation between variables was calculated using the Spearman rank correlation analysis. *P* < 0.05 was considered statistically significant. All analyses were performed using SPSS 17.0 Inc. (IBM).

## Results

### Description of the individuals followed in this study

We evaluated 39 individuals divided into three groups: (i) Thirteen VL individuals, HIV^+^ individuals that did not receive antiretroviral treatment and presented with detectable VL in plasma, including 11 males and two females (age: 36.0 years [25.8–50.5], median [Q1-Q3]. Plasma VL 6.31*10^4^ [10^3^−1.26*10^6^] copies/ml; median [Q1-Q3]); (ii) Fourteen uVL individuals, HIV^+^ individuals that received antiretroviral treatment and presented with uVL in plasma (<50 copies/mL), including 13 males and one female (age: 41.5 years [35.0–53.0], median [Q1-Q3]); and (iii) twelve uninfected healthy individuals (controls), including 9 males and three females (age: 39.0 [24.0–46.5] years, median [Q1-Q3]). The three groups were similar in age and gender (Table 1). VL and uVL-HIV^+^ individuals showed the same level of nadir CD4^+^ T-cell counts (380.8 [66.7–517.2] cells/μl and 244.5 [122.6–349.2] cells/μl [Q1-Q3], respectively, *p* = 0.681) and percentage of nadir CD4^+^ T-cells (19.6% [9.5–30.7] and 14.5% [8.9–19.9], respectively, *p* = 0.301). VL-HIV^+^ individuals were recruited at the time of diagnosis just before starting an antiretroviral treatment.

### Immune cells populations in HIV^+^ individuals and healthy individuals

We first investigated the different immune cell population frequencies in HIV^+^ and healthy individuals (S1 Fig). Total B cells were significantly decreased in VL-HIV^+^ individuals in comparison with healthy individuals. The frequency of CD4^+^ T cells significantly decreased, but the frequency of CD8^+^ T cells increased in VL-HIV^+^ individuals. These frequencies induced an inversion in the CD4/CD8 ratio in HIV^+^ individuals (Table 1). The CD4/CD8 ratio is related to immune system exhaustion in HIV^+^ infected individuals. It was interesting to note that in effectively anti-HIV-treated uVL-individuals, this ratio was still < 1, showing that even with undetectable levels of HIV for over three years, the immune system appeared to be dysregulated (Table 1). In CD4^+^ and CD8^+^ T cells, cellular subsets presented an activated status that was significantly increased in VL-HIV^+^ individuals. In summary, as expected, the immune system presented high expressions of activation markers in treatment-naïve HIV-1^+^ individuals (VL). However, not all of these markers returned to the normal levels found in healthy individuals after anti-HIV treatment, showing that immune system dysregulation persisted even in absence of a replicative virus.

### Expression of PD-1 and PD-L1 markers on T and B cells

**Fig 1:**
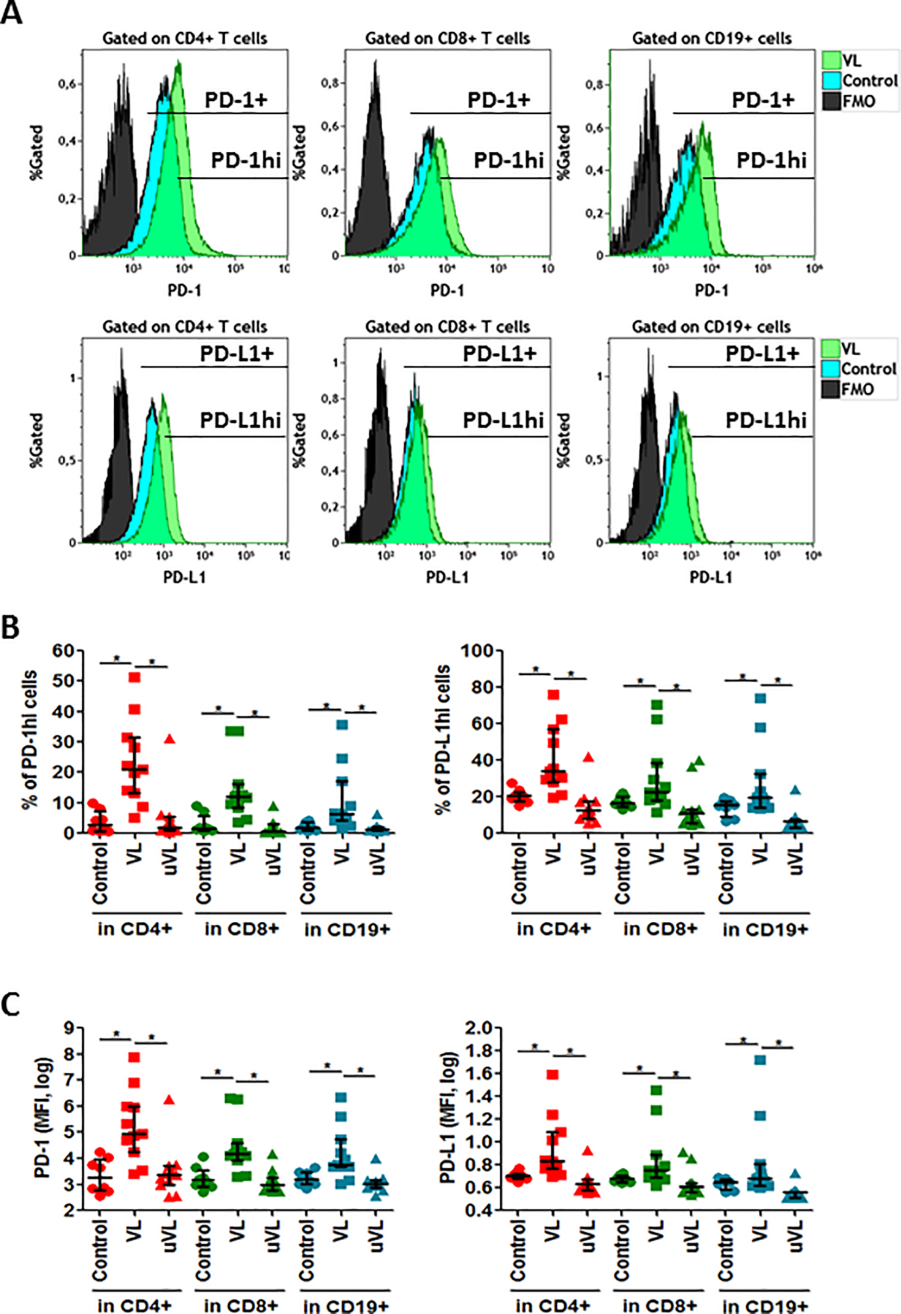
PD-1 and PD-L1 expression. Whole blood was labeled to determine PD-1 and PD-L1 subsets. (A) Histogram plots representatives of one healthy individual and one VL-HIV-1^+^ individual. PD-L1^+^ and PD-L1^hi^ were determined in CD4^+^, in CD8^+^ and CD19^+^ cells. (B) Frequencies of PD-1^hi^ or PD-L1^hi^ in CD4^+^, CD8^+^ T and CD19^+^ cells and (C) MFI (median fluorescence intensity) of PD-1 and PD-L1 expression in CD4^+^, CD8^+^ T and CD19^+^ cells. Median [Q1-Q3]; represented. * = *p*<0.05 when comparing conditions. Each symbol corresponds to an individual.

During HIV-1 infection, weak antiviral immune responses lead to chronic infections that in turn induce the activation of negative regulatory pathways, such as the PD-1/PDL-1 axis. Therefore, we analyzed PD-1 and PD-L1 marker expression on several cellular subsets such as CD8^+^ and CD4^+^ T cells and on CD19^+^ cells (B cells). Both markers were highly expressed on T and B cells (Fig 1A and S2 Fig). Thus, we followed PD-1^hi^ and PD-L1^hi^ expressions along with the expression intensities at the surface of T and B cells from HIV^+^ and healthy individuals (Fig 1A). The frequencies of PD-1^hi^ and PD-L1^hi^ (Fig 1B) had significantly increased in CD4^+^ and CD8^+^ T cells and CD19^+^ B cells as the mean fluorescent intensities (MFI) of PD-1 and PD-L1 expressions on the three cellular compartments (Fig 1C). The effective treatment in uVL-HIV^+^ individuals reduced the MFI to the levels observed in healthy individuals. Therefore, we confirmed that PD-1 and PD-L1 were over-expressed at the surface of T and B cells during HIV-1 replication.

**Fig 2:**
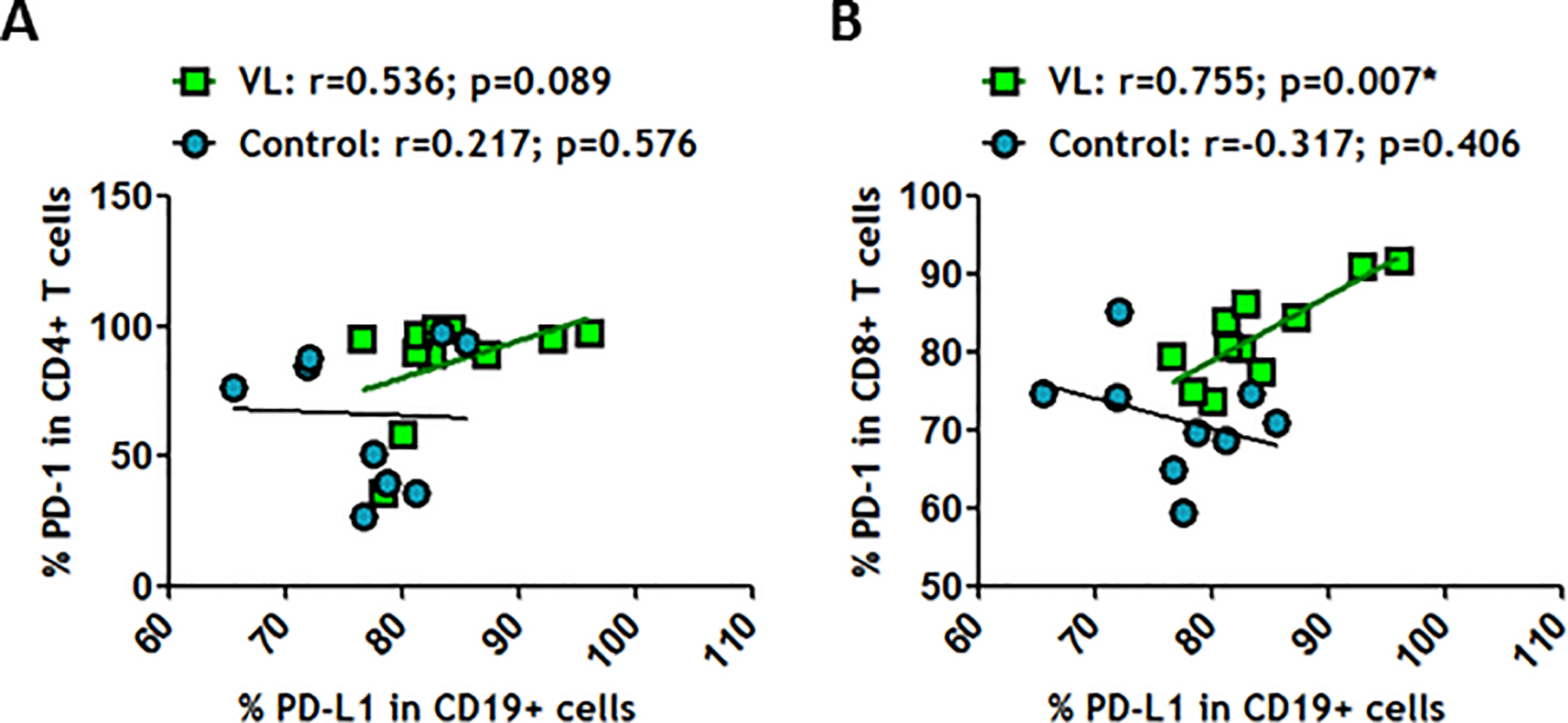
PD-L1-expressing CD19^+^ correlated positively with PD-1-expressing CD8^+^ T cells in HIV-1^+^ individuals. (A) Correlation between frequencies of PD-L1 in CD19^+^ cells and PD-1 in CD4^+^ T cells and (B) Correlation between frequencies of PD-L1 in CD19^+^ cells and PD-1 in CD8^+^ T cells were determined in healthy individuals and in VL-HIV-1^+^ individuals. Correlations were determined by Spearman’s rank correlation. * = *p*<0.05 when comparing conditions. Each symbol corresponds to an individual.

Since the union between PD-1 and its ligand, PD-L1, is pivotal to blocking cell activation, we analyzed the relationships between both markers. No correlation was observed between CD19^+^PD-L1^+^ and CD4^+^PD-1^+^ in VL-HIV-1^+^ and healthy individuals (Fig 2A). However, a positive correlation between CD19^+^PD-L1^+^ and CD8^+^PD-1^+^ only in VL-HIV-1^+^ individuals was observed (Fig 2B). This outcome may help us to understand why HIV-specific CD8^+^ T cells are so strongly impaired during HIV-1 infection. In summary, these results showed an imbalance in PD-1/PD-L1 axis in T and B-cell compartments in VL-HIV-1^+^ individuals in comparison to uVL-HIV-1^+^ and healthy individuals, which could explain the lack of efficient antiviral function during HIV-1 disease.

### CD72/CD100 expression on T and B-cell surface

**Fig 3:**
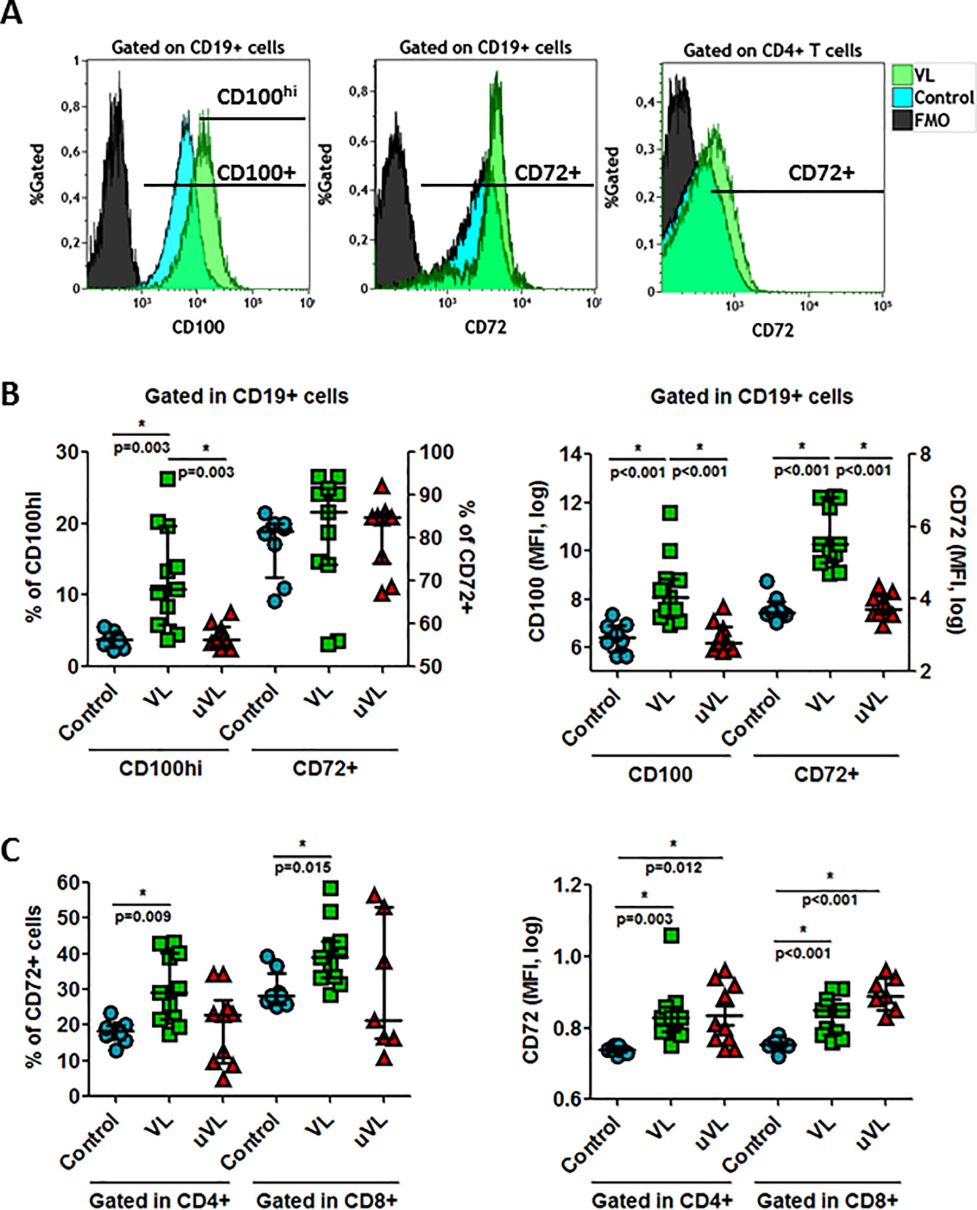
CD100 and CD72 expression in T (CD4^+^ and CD8^+^ T cells) and CD19^+^ cells. Whole blood was labeled to determine CD72 and CD100 expression. (A) Histogram plots representatives of one healthy individual and VL-HIV-1^+^ individuals. (B) Frequencies of CD100^hi^ and CD72 and median of fluorescent intensity (MFI) of CD100 and CD72 gated on CD19^+^ cells. (C) Frequencies of CD72 and MFI of CD72 presents at the surface of CD4^+^ and CD8^+^ T cells. Median [Q1-Q3]; represented. * = *p*<0.05 when comparing conditions. Each symbol corresponds to an individual.

The CD72 marker, which is a negative regulator of B cell responsiveness by preventing the differentiation of naïve B cells to plasmablasts, and has been poorly studied in the HIV-1 context, was analyzed (S3 Fig). CD72 is the receptor for the class IV semaphorin (CD100) that is also highly expressed at the surface of B cells (Fig 3A) and at the surface of T cells (*data not shown*). No significant changes were observed in the frequencies and in the MFI of CD100 at the surfaces of CD4^+^ and CD8^+^ T cells (*data not shown*). On the contrary, CD72 and CD100^hi^ expression levels were increased at the surface of B cells in HIV-1^+^ individuals (Fig 3A and 3B) even if frequencies of CD72^+^ B cells did not present significant changes (Fig 3B). In addition, we observed an increase in CD72 frequencies and MFI at the surface of CD4^+^ and CD8^+^ T cells (Fig 3C) in VL-HIV-1^+^ individuals. It was interesting to note that CD72 MFI at the CD4^+^ and CD8^+^ T cell surfaces were identical in the two groups of analyzed HIV-1^+^ individuals (Fig 3C), showing that effective treatment did not reverse the increase in CD72 MFI. In conclusion, we observed an increase in CD100 expression at the surface of B cells and a parallel increase in the expression of its receptor, CD72, on B and T cells from HIV-1^+^ individuals.

### CD100 frequency is associated with PD-1 and PD-L1 expression on T cells

**Fig 4:**
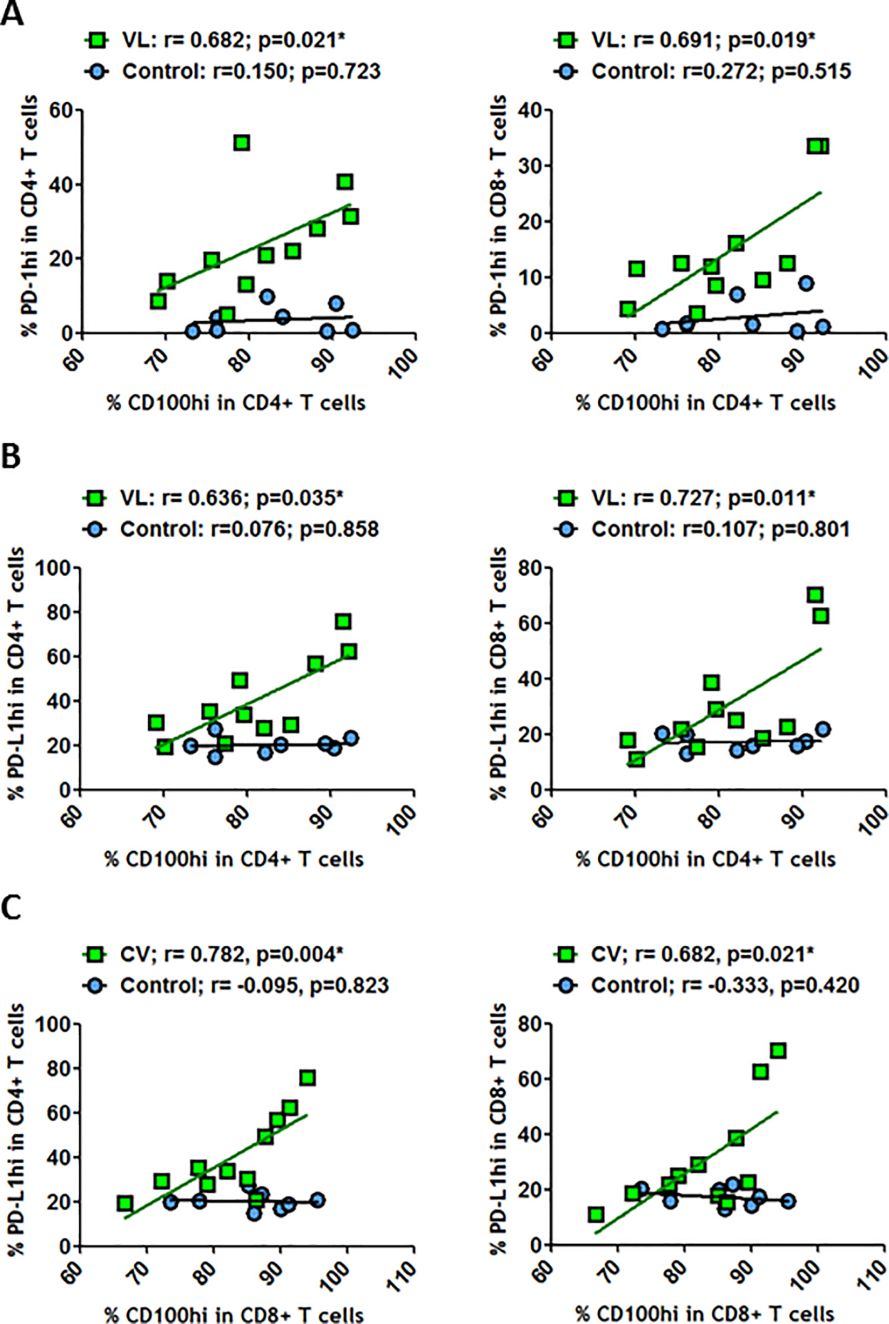
Correlations between CD100^hi^ and PD-1^hi^ or PD-L1^hi^ on T or B cells. (A) Correlations between frequency of CD100^hi^ on CD4^+^ T cells and frequencies of PD-1^hi^ on CD4^+^ T cells and on CD8^+^ T cells. (B) Correlations between frequencies of CD100^hi^ on CD4^+^ T cells and frequencies of PD-L1^hi^ on CD4^+^ T cells and on CD8^+^ T cells. (C) Correlations between frequencies of CD100^hi^ on CD8^+^ T cells and frequencies of PD-L1^hi^ on CD4^+^ T cells and on CD8^+^ T cells. All these correlations were determined in healthy individuals and in VL-HIV-1^+^ individuals. Correlations were determined by Spearman’s rank correlation. * = *p*<0.05 when comparing conditions. Each symbol corresponds to an individual.

**Fig 5:**
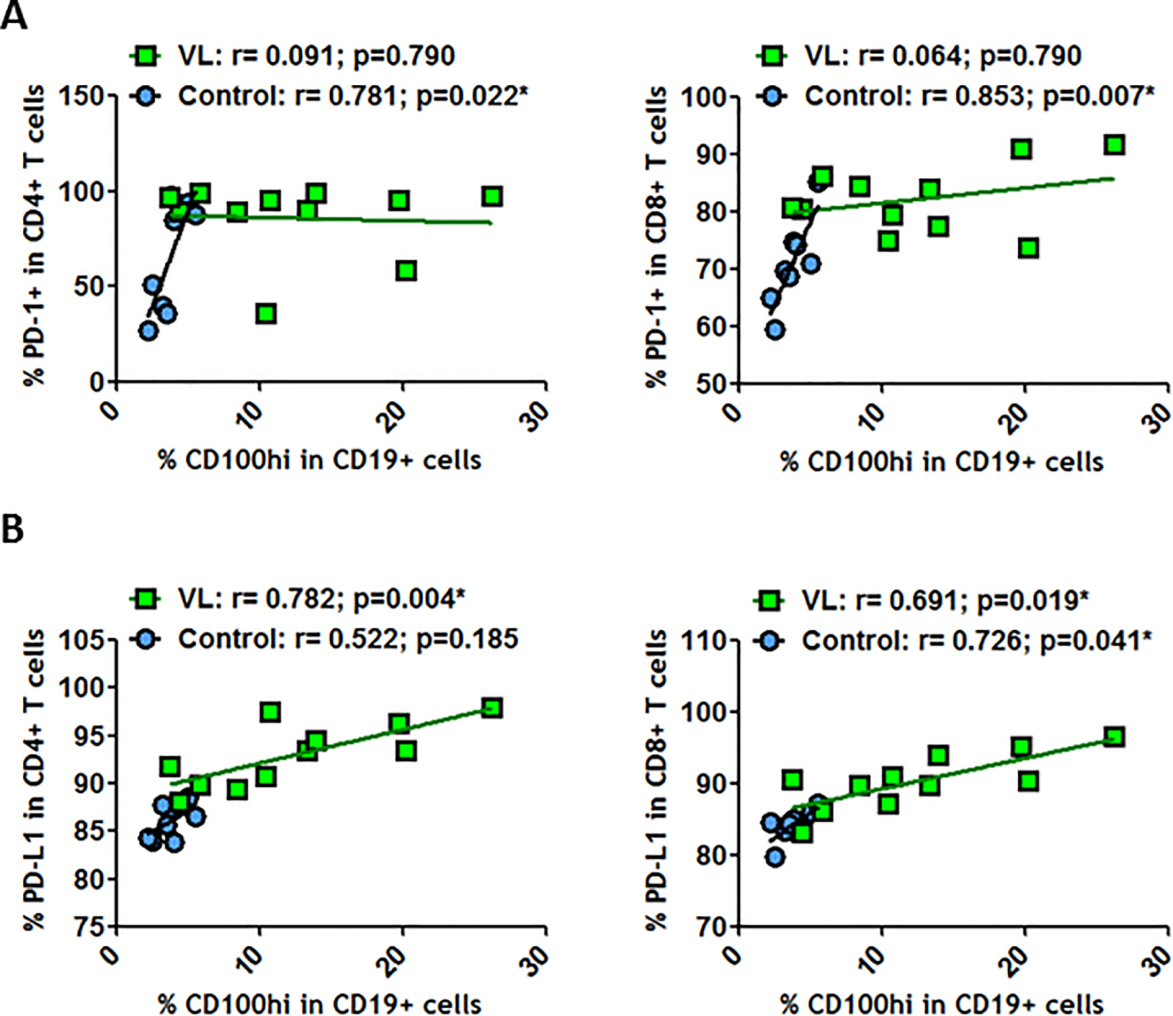
Correlations between CD100^hi^ on CD19^+^ cells and PD-1 and PD-L1 on T cells. (A) Correlations between frequencies of CD100^hi^ on CD19^+^ cells and frequencies of PD-1 on CD4^+^ T cells and on CD8^+^ T cells. (B) Correlations between frequencies of CD100^hi^ on CD19^+^ cells and frequencies of PD-L1 on CD4^+^ T cells and on CD8^+^ T cells. Correlations were determined by Spearman’s rank correlation. * = p<0.05 when comparing conditions. Each symbol corresponds to an individual.

As there was a general increase in PD-1, PD-L1, CD72 and CD100 expressions at the surface of T and B cells, we analyzed whether any of these markers were specifically correlated. No relationships were observed between CD100^hi^ expressed at the surface of CD4^+^ T cells and PD-1^hi^ at the surface of CD4^+^ and CD8^+^ T cells in healthy individuals (Fig 4A). However, positive and significant correlations were observed in VL-HIV-1^+^ individuals (Fig 4A). Such positive correlations were also observed between CD100^hi^ levels on CD4^+^ T and CD8^+^ T cells and PD-L1^hi^ expressed on CD4^+^ and CD8^+^ T cells (Fig 4B and 4C) but not in healthy individuals. Therefore, during HIV-1 replication, CD100 expression was related to the PD-1/PD-L1 axis. On the other hand, we observed that CD100 expression levels were also increased at the surface of CD19^+^ cells (Fig 3B). Thus, we studied the relationship between CD19^+^CD100^hi^ and the PD-1/PD-L1 axis. We observed that CD19^+^CD100^hi^ was positively and significantly correlated with PD-1 expression at CD4^+^ and CD8^+^ T cell surfaces in healthy individuals (Fig 5A). These correlations were not observed during HIV-1 replication. In contrast, CD19^+^CD100^hi^ showed significant correlation with CD8^+^PD-L1^+^ and CD4^+^PD-L1^+^ in VL-HIV-1^+^ individuals (Fig 5B). Therefore, the increase in the activation signal CD100 at the surface of B cells was associated with the PD-L1 marker that appears to be related to viral escape during HIV-1 infection.

**Fig 6:**
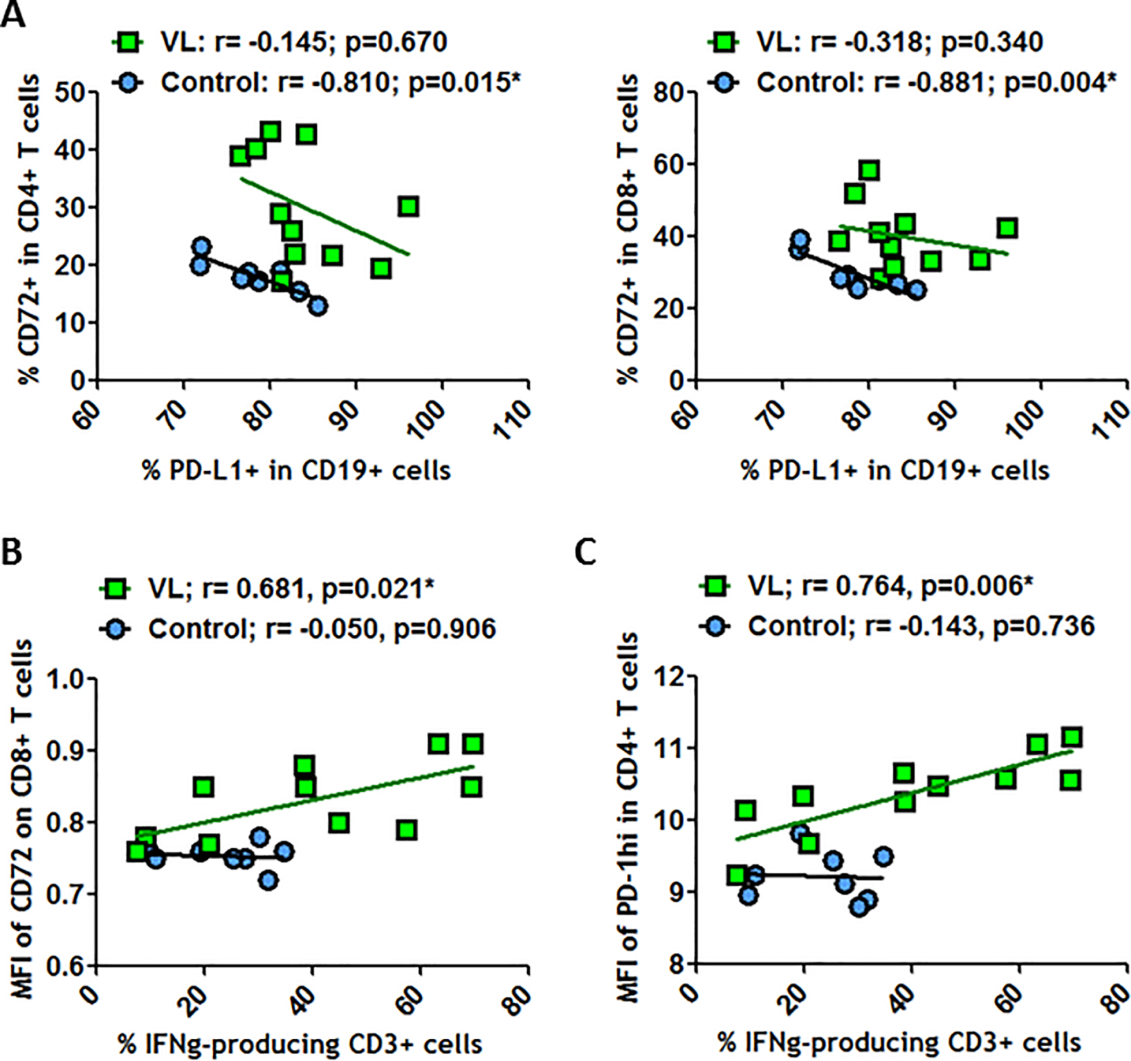
Correlations between PD-L1^+^ on CD19^+^ cells and CD72^+^ on T cells. (A) Correlations between frequencies of PD-L1 on CD19^+^ cells and frequencies of CD72 on CD4^+^ T cells and on CD8^+^ T cells. (B) Correlations between frequencies of IFN-γ-producing CD3^+^ T cells and MFI of CD72 on CD8^+^ T cells. (C) Correlations between frequencies of IFN-γ-producing CD3^+^ T cells and MFI of PD-1^hi^ on CD4^+^ T cells. All these correlations were determined in healthy individuals and in VL-HIV-1^+^ individuals. Correlations were determined by Spearman’s rank correlation. * = *p*<0.05 when comparing conditions. Each symbol corresponds to an individual.

On the other hand, CD72 expressed on CD4^+^ and CD8^+^ T cells was significantly and negatively correlated with PD-L1 at B cell surfaces in healthy individuals (Fig 6A). This finding confirmed a link between both suppressive markers CD72 and PD-L1 and indicated that CD72/CD100 and PD-1/PD-L1 axes have a role in immune homeostasis. Nevertheless, during HIV-1^+^ infection, these relationships were in part lost.

Because CD100 expression on CD8^+^ T cells was observed to be related to IFN-γ production by peripheral blood mononuclear cells (PBMC) during HCV infection [30], we analyzed the relationship between CD72/CD100 axis and IFN-γ-producing CD3^+^ T cells in the HIV-1 context. No correlation was observed between CD100-expressing T cells (CD4^+^ or CD8^+^ T cells) and IFN-γ production (*data not shown*). However, the MFI of CD72 expressed on CD8^+^ T cells was clearly and positively correlated with IFN-γ-producing CD3^+^ T cells in VL-HIV-1^+^ individuals but not in uVL-HIV-1^+^ or healthy individuals (Fig 6B and *data not shown*). Similarly, a positive correlation was observed between the MFI of the PD-1^hi^ expression on CD4^+^ T cells and IFN-γ-producing T cells (Fig 6C). Therefore, not only imbalances associated with the upregulation of studied markers in T and B cells during HIV-1^+^ infection, but also the dysregulation of relationship between the costimulatory markers CD72 and PD-1 and the production of a pro-inflammatory cytokine on T cells were observed, which may be a very important part of the dysregulation of immune system homeostasis and hyper-activation.

## Discussion

**Fig 7:**
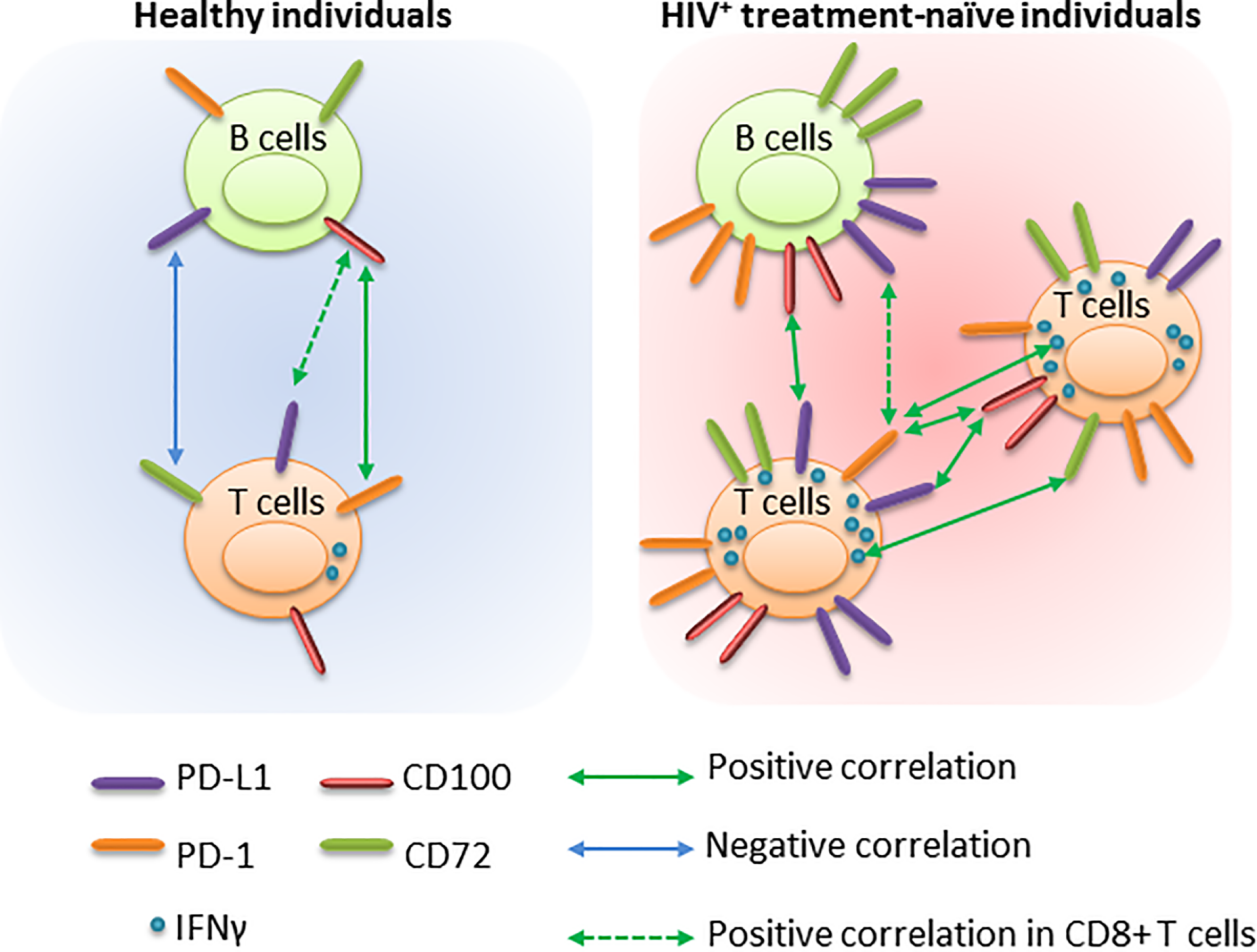
Summary of the potential relationships between B and T cells in healthy and in HIV^+^ treatment-naïve individuals.

In this study, we report that treatment-naïve HIV-1 infected individuals present with an increase in PD-1/PD-L1 and CD72/CD100 axis markers when compared to patients on antiretroviral therapy or healthy controls (summarized in Fig 7).

Efficient antiretroviral treatment permitted a reversion of such high expression, except for the levels of CD72 expression at CD4^+^ and CD8^+^ T cell surfaces. This finding indicated that a replicative virus was generally needed to induce up-regulation of these markers. CD72 was already observed to be increased after cell activation [22]. Thus, sustained CD72 expression at CD4^+^ and CD8^+^ T cell surfaces might be linked to the maintenance of general activation of the immune system observed in HIV^+^ individuals. This deduction was supported by the association observed between CD72 expression levels and the frequency of IFNγ-producing T cells. Therefore, we hypothesized that CD72/CD100 may be another exhaustion marker presented by the immune system. One of the markers of the immune dysfunction is a blunted T cell signaling response in HIV^+^ progressors, which was predominantly related to a high basal intracellular phosphorylation level [31]. General phosphorylation is associated with cellular activation, but on other hand constant phosphorylation of CD72 negatively regulates B-cell receptor (BCR) signaling on B cells [32], and phosphorylation of PD-1 might inhibit T-cell receptor (TCR) and BCR signaling on T and B cells, respectively [33]. On other hand, CD72 on T cells is an activation marker [22], and its phosphorylation might maintain a TCR-related activation state. This sustained phosphorylation level in immune cells might be pivotal in understanding hyper-activation and therefore in understanding immune system exhaustion.

More interestingly, a relationship between CD100^+^ (a marker related to B-cell activation [28, 29]) on CD19^+^ cells and PD-L1 on CD4^+^ and CD8^+^ T cells on VL-HIV-1^+^ individuals was observed (Fig 7). Such positive correlations were not detected in healthy individuals. The association between proliferating B cells and suppressive CD4/CD8 T cells provides an indication of a dysregulated HIV-specific response. Also, this dysregulation may be observed with the loss of the positive correlation between CD19^+^CD100^hi^ and the exhaustion marker PD-1 expression at the surface of CD4^+^ and CD8^+^ T cells in VL-HIV-1^+^ individuals (Fig 7). This loss of correlation permitted us to confirm that the immune system was not able to control the balance between cellular proliferation and activation/exhaustion. In parallel, a negative correlation between CD19^+^PD-L1^+^ and CD72-expressing CD4^+^ and CD8^+^ T cells in healthy individuals was observed and may be related to finely tuned control of activation and proliferation status in healthy individuals but not in VL-HIV-1^+^ individuals. Indeed, CD72/CD100 axis on T-cell subsets is associated with T cell activation and proliferation. In addition, CD72 has been shown to be involved in the expansion of the regulatory T cells [22, 23]. We assume that the contact between CD19^+^PD-L1^+^ and activated T cells may limit inflammation in the normal immune system in healthy individuals, which can be lost during HIV-1 infection. Very little is known about the role of CD72 at the B and T cell surface, but its union with CD100 has been shown to be associated with the activation of the immune regulation [24, 30]. Loss of suppression of the immune system may induce hyper-activation leading further to cellular exhaustion. Our results seem to confirm this hypothesis since the CD100 increases at CD4^+^ and CD8^+^ T cell surfaces were associated with PD-1 and PD-L1 expression increases CD4^+^ and CD8^+^ T cell surfaces. This data indicates that the CD100, an activation marker, is associated with markers of T cell suppression, namely PD-1 and PD-L1. It has already been showed that CD100 is important for antigen-specific responses since HIV-1–specific IFN-γ production is associated with the absolute number of CD100^+^CD8^+^ T cells [19, 30] and that PD-1 is associated with immune dysfunction [6, 7]. During HCV infection, the increase in CD100 at the surface of naïve CD8^+^ T cells interacting with the CD72 receptor was related to an enhanced PBMC capability to secrete IFN-γ and TNF-α [30]. In our study, we showed a correlation between the level of CD72 expression on CD8^+^ T cells and the frequency of IFN-γ-producing T cells. Therefore, it seems that CD72 on B cells may have a suppressive function but may be linked to a pro-inflammatory response when expressed on T cells [34–36]. We have previously demonstrated that HIV-1 could direct B cells to an IL-10-producing phenotype, which could as a consequence, contribute to immune system dysregulation [37]. These data, together with findings from this study, highlight the potential dual functionality of the CD72/CD100 axis to first maximize immune system activation and second to mediate immune suppression via IL-10 production. Additional investigations will be required to confirm such hypothesis in the context of HIV-1 infection.

On the other hand, it has previously been reported that expansion of CD8^+^ T cells lacking CD100 was observed in HIV-1 infection [19] although an increase in CD8^+^-expressing CD100 was demonstrated in our work. However, one conclusion matches Eriksson’s work, in which it was demonstrated that CD100 expression on T cells seems to be associated with HIV-1 disease progression. It would be interesting to examine such opposite results in future since the CD100/CD72 axis might be very important for immune activation regulation. In our work, we observed that CD8^+^CD100^+^ T cells were positively correlated with both PD-L1^+^CD4^+^ and PD-L1^+^CD8^+^ T cells, which may explain why a reduced proliferative ability and reduced effector function of CD100^+^CD8^+^ T cells were observed in HIV-1^+^ individuals.

It was described that activation of T cells could lead to the cleavage of CD100, which permitted the increase of soluble CD100 in plasma [38] and was positively correlated with disease severity or progression in various pathologies [39–41]. However, it has been shown that soluble CD100 in plasma had decreased in HIV-1^+^ individuals [42], and we demonstrated that CD100 expression was increased at the surface of B cells and did not change at T cell surface. Therefore, we hypothesized a lack of cleavage of the CD100 protein, and as a consequence, could lead to a sustained or higher presence of CD100 at T and B cell surfaces. Moreover, the presence of high CD100 levels at the cell surface could be associated with improved interactions with CD72-bearing cells, which could lead to an increase in intracellular phosphorylation and B-cell dysregulation [43, 44].

Immune system dysfunction during HIV-1 infection is undoubtedly multifactorial, but accumulating evidence indicates that CD100 and CD72 may play an important role in immune dysregulation of effector cell functions [19–21]. It would be interesting to contrast CD100 and CD72 expression markers with the others negative checkpoint regulators (NCR) such as LAG-3, TIM-3, 2B4, or CTLA-4 on T cells, NK, and DC [10]. Indeed, it was demonstrated that PD-1 and PD-L1 are both members of the NCR family; cells expressing these both markers (on NK or T cells) and able to express also LAG-3 and TIM3 were even more exhausted than cells expressing only PD-1 [45]. Moreover, it was demonstrated that invariant NK T cells, which are important immunoregulatory cells that can influence other immune cells, can express high 2B4 levels during HIV infection and that high 2B4 expression is correlated with HIV disease progression [46]. Therefore, it will be highly interesting to analyze CD72/CD100 and PD-1/PD-L1 expressions in the context of other NCR expression in order to obtain a general vision of cellular exhaustion during HIV infection.

Future studies with larger samples sizes and more female patients might confirm these hypotheses. Indeed, the modest samples size it is one of the limitations of our study and in addition the population studied was predominantly male (>75%) so generalizability to female should be also applied cautiously.

To our knowledge, no group has demonstrated that CD100 and CD72 and the PD-1/PD-L1 axis were associated during HIV-1 infection. We hypothesized that there are two indications that may be associated with upregulation of the expression of all these markers and may limit adequate immune system responses thus leading it to hyper-activation and exhaustion. One indication suggests that the PD-1/PD-L1 interaction may exert inhibitory effects that limit effector cells responses. The second indication is based on the observed increase in CD72/CD100, which might maximize the T- and B-cells activation and suggests that it might also be involved in limiting immune system response. Based on the data reported in this study, it is suggested that further studies should be performed to investigate the role of the CD72/CD100 axis upon T- and B-cell exhaustion.

## Supporting information

S1 Table Antibodies and fluorochromes used in this study. (PPTX)

S1 Fig Dot plots defining cellular subsets. (A)Whole blood was labeled to determine the frequency of CD4^+^ T and CD8^+^ T cells (gated on lymphocytes population). (B) Naïve cells were determined as CD45RA^+^CD27^+^; Activated subsets were determined as CD45RO^+^/HLA-DR^+^ and Memory subsets were determined as CD45RO^+^. (C) Whole blood was labeled to determine the frequency of B cells (CD19^+^, gated on lymphocytes population), Memory (CD27^+^); Naïve (IgD^+^CD27^-^) and plasmablasts (CD24^-^CD38^high^) were determined in total B cells population. (D) PBMC isolated by Ficoll gradient were labeled for surface markers and further intracellularly for IFNγ. IFNγ-producing cells were gated on viable CD3+ T cells. Dot plots from one donor are shown.
(PPTX)

S2 Fig Dot plots displaying gating strategy to define PD-1 and PD-L1 subsets. (A)Whole blood was labeled to determine the frequency of CD4^+^ T, CD8^+^ T cells and CD19^+^ B cells (gated on lymphocytes population). (B) CD4^+^ T, CD8^+^ T cells and CD19^+^-expressing PD-1 and PD-L1 were analyzed using the Fluorescence Minus One (FMO) gating strategy. Dot plots from one donor are shown.
(PPTX)

S3 Fig Dot plots displaying gating strategy to define CD72 and CD100 subsets. Whole blood was labeled to determine the frequency of CD72 and CD100-expressing CD4^+^ T, CD8^+^ T cells and CD19^+^ B cells (gated on lymphocytes population). Dot plots from one donor are shown.
(PPTX)
