# Supplementary figures and images for: CD72/CD100 and PD-1/PD-L1 markers are increased on T and B cells in HIV-1+ viremic individuals, and CD72/CD100 axis is correlated with T-cell exhaustion
Source: PLoS One. 2018 Aug 30;13(8):e0203419. doi: 10.1371/journal.pone.0203419 (PMC6117071; doi:10.1371/journal.pone.0203419)

## Slide 1
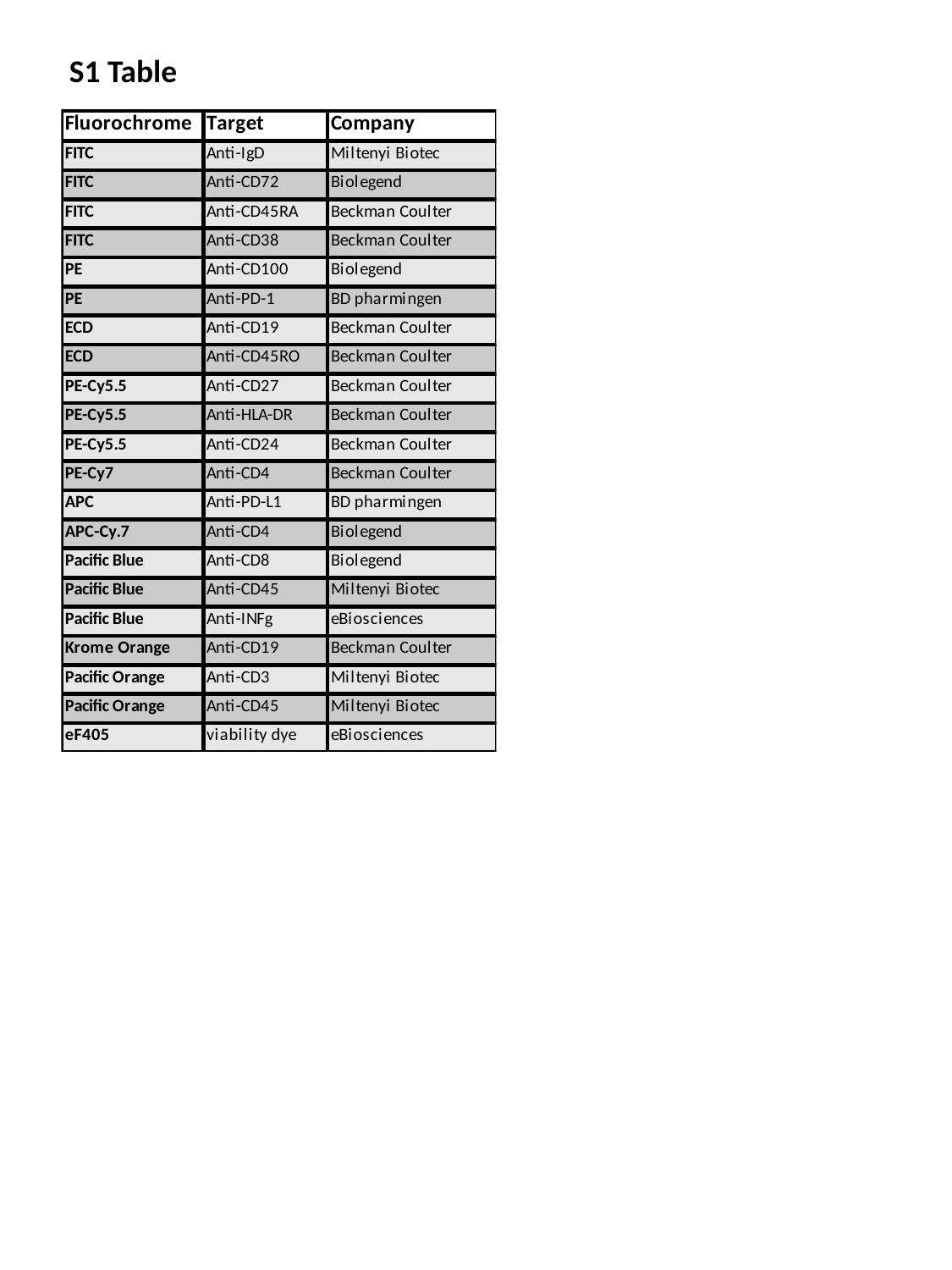

S1 Table

Supplement: S1 Table — (PPTX) [file pone.0203419.s001.pptx]

## Slide 1
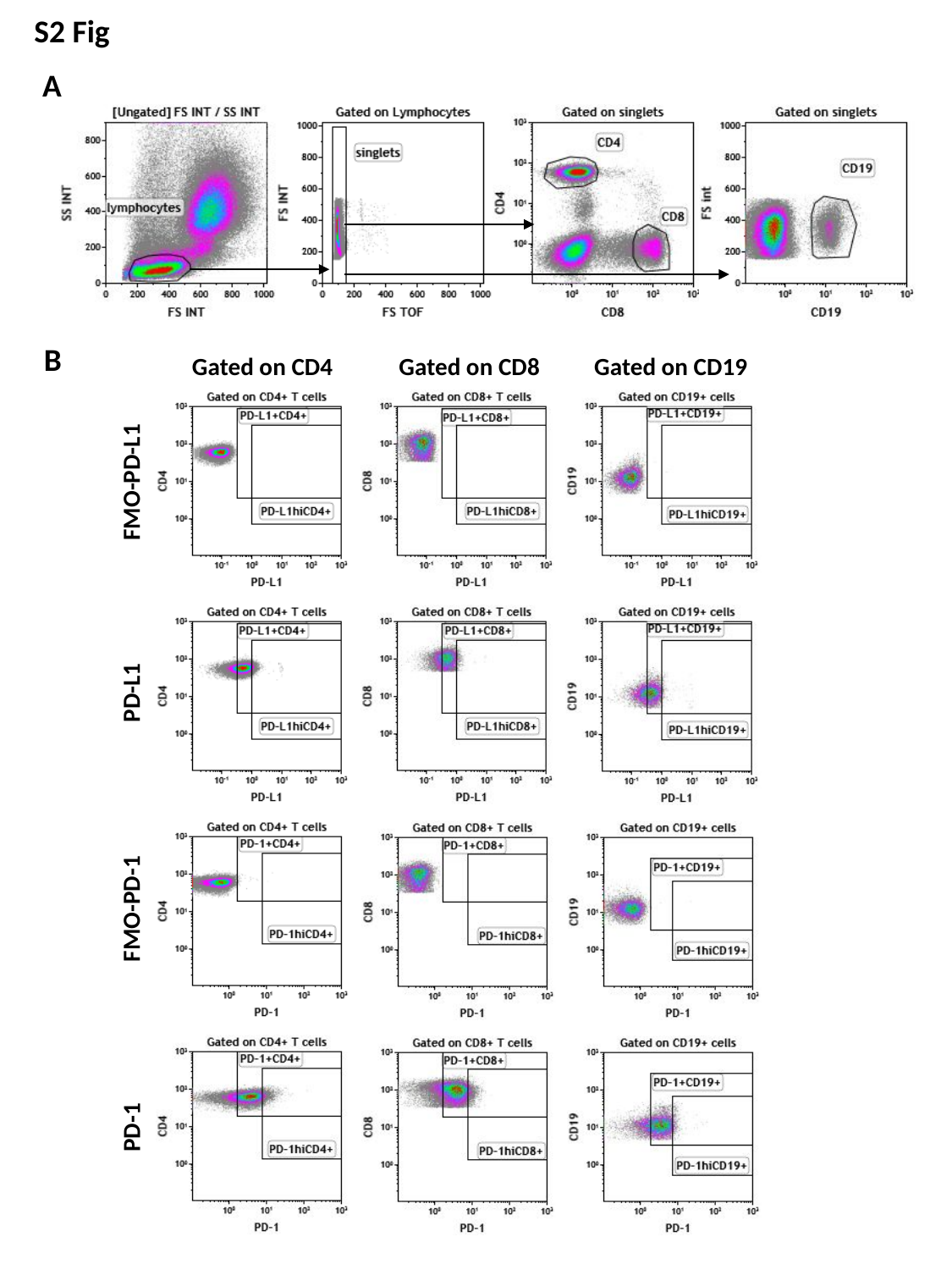

S2 Fig
A
B
Gated on CD4
Gated on CD8
Gated on CD19
FMO-PD-L1
PD-L1
FMO-PD-1
PD-1

Supplement: S2 Fig — (A)Whole blood was labeled to determine the frequency of CD4+ T, CD8+ T cells and CD19+ B cells (gated on lymphocytes population). (B) CD4+ T, CD8+ T cells and CD19+-expressing PD-1 and PD-L1 were analyzed using the Fluorescence Minus One (FMO) gating strategy. Dot plots from one donor are shown. (PPTX) [file pone.0203419.s003.pptx]

## Slide 1
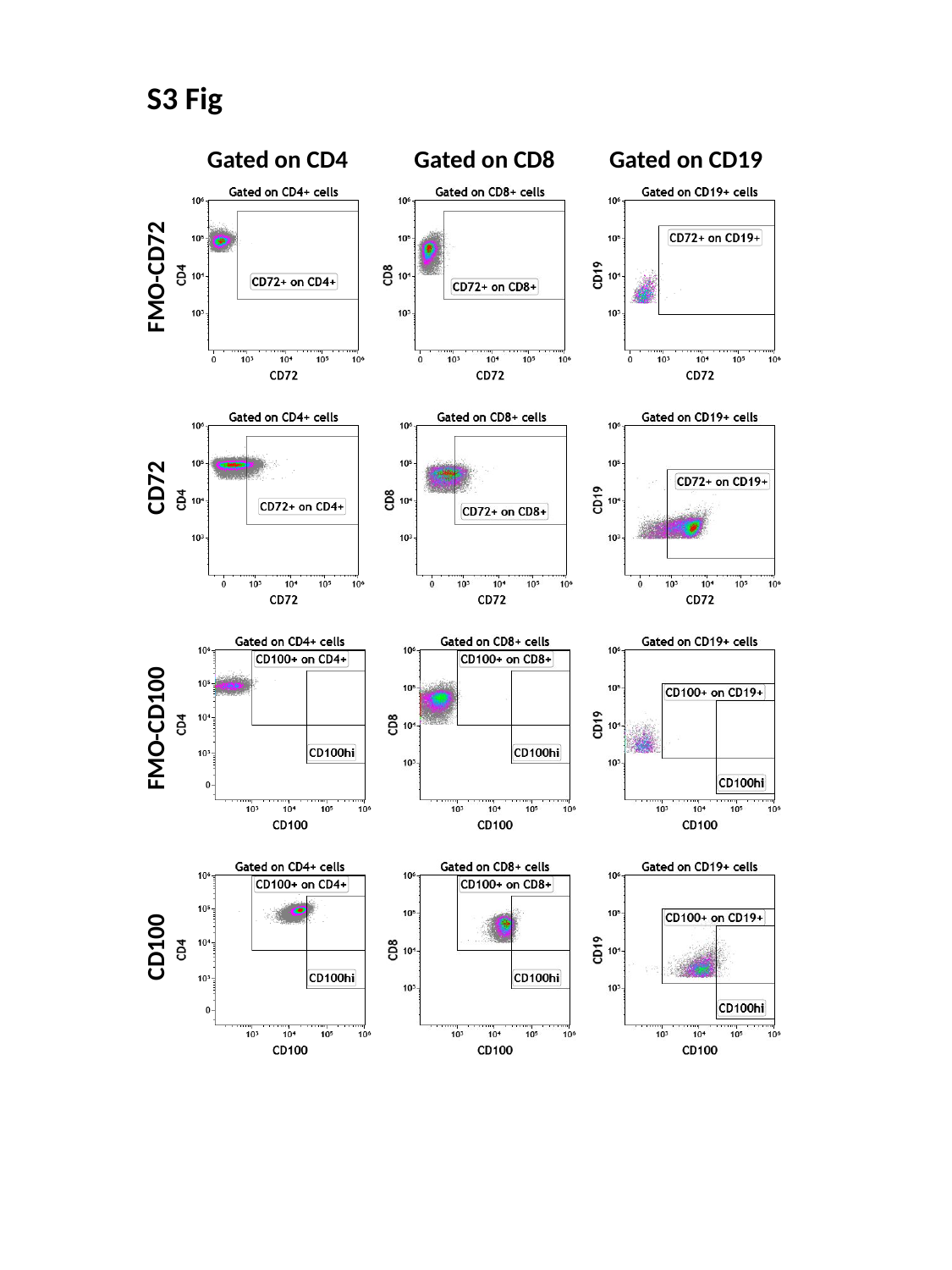

S3 Fig
Gated on CD4
Gated on CD8
Gated on CD19
FMO-CD72
CD72
FMO-CD100
CD100

Supplement: S3 Fig — Whole blood was labeled to determine the frequency of CD72 and CD100-expressing CD4+ T, CD8+ T cells and CD19+ B cells (gated on lymphocytes population). Dot plots from one donor are shown. (PPTX) [file pone.0203419.s004.pptx]
