# Supplementary material for: CD72/CD100 and PD-1/PD-L1 markers are increased on T and B cells in HIV-1+ viremic individuals, and CD72/CD100 axis is correlated with T-cell exhaustion
Source: PLoS One. 2018 Aug 30;13(8):e0203419. doi: 10.1371/journal.pone.0203419 (PMC6117071; doi:10.1371/journal.pone.0203419)
Supplement: S1 Fig — (A)Whole blood was labeled to determine the frequency of CD4+ T and CD8+ T cells (gated on lymphocytes population). (B) Naïve cells were determined as CD45RA+CD27+; Activated subsets were determined as CD45RO+/HLA-DR+ and Memory subsets were determined as CD45RO+. (C) Whole blood was labeled to determine the frequency of B cells (CD19+, gated on lymphocytes population), Memory (CD27+); Naïve (IgD+CD27-) and plasmablasts (CD24-CD38high) were determined in total B cells population. (D) PBMC isolated by Ficoll gradient were labeled for surface markers and further intracellularly for IFNγ. IFNγ-producing cells were gated on viable CD3+ T cells. Dot plots from one donor are shown. (PPTX) [file pone.0203419.s002.pptx]

## Slide 1
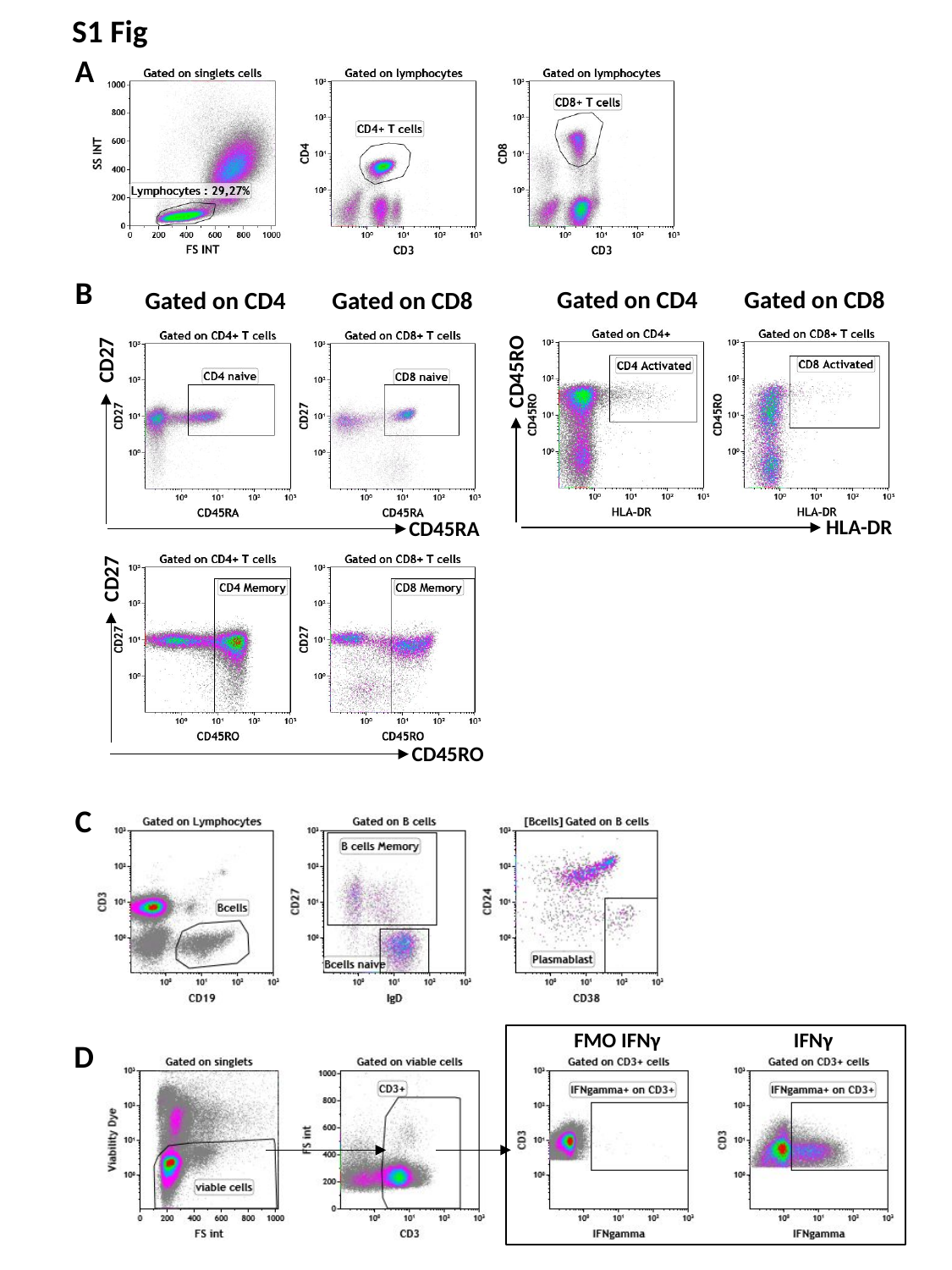

S1 Fig
A
B
Gated on CD4
Gated on CD8
Gated on CD4
Gated on CD8
CD27
CD45RO
HLA-DR
CD45RA
CD27
CD45RO
C
FMO IFNγ
IFNγ
D
